# Age-Related Decline of Neutrophilic Inflammation Is Associated with Better Postoperative Prognosis in Non-eosinophilic Nasal Polyps

**DOI:** 10.1371/journal.pone.0148442

**Published:** 2016-02-05

**Authors:** Dae Woo Kim, Dong-Kyu Kim, Ara Jo, Hong Ryul Jin, Kyoung Mi Eun, Ji-Hun Mo, Seong H. Cho

**Affiliations:** 1 Department of Otorhinolaryngology-Head and Neck Surgery, Boramae Medical Center, Seoul National University College of Medicine, Seoul, Korea; 2 Division of Allergy-Immunology, Department of Internal Medicine, University of South Florida College of Medicine, Tampa, Florida, United States of America; 3 Department of Otorhinolaryngology-Head and Neck Surgery, Chuncheon Sacred Heart Hospital and Nano-Bio Regenerative Medical Institute, Hallym University College of Medicine, Chuncheon, Korea; 4 Department of Otorhinolaryngology, Dankook University College of Medicine, Chonan, Korea; University of Gdansk, POLAND

## Abstract

**Background:**

Innate and adaptive immune responses change with increasing age and affect the course of diseases. Previous study investigated immunologic alteration in Western nasal polyps (NP) which is mostly eosinophilic. However, there are no reports regarding age-related immune changes of non-eosinophilic NP (NE-NP) which is a predominant subtype in Asian population.

**Methods:**

A total of 153 subjects, including 20 with control, 63 with chronic rhinosinusitis (CRS) without NP (CRSsNP), and 70 with CRS with NP were enrolled. Age-related changes in computed tomography (CT), cytokines and clinical information were investigated. Tissue samples were analyzed for protein levels of IL-5, IL-17A, IL-23, interferon (IFN)-γ, CCL-11, and CXCL-8, using Luminex immunoassay and for mRNA expression levels of interleukin (IL)-5, IL-17A, IL-23p19, IFN-γ, CCL-11, CXCL-1, CXCL-2, CXCL-8, and CXCR2 by quantitative RT-PCR. Immunohistochemistry (IHC) was performed for the number of inflammatory cells.

**Results:**

We observed that Lund-Mackay CT scores decreased with age in NE-NP. The number of human neutrophil elastase-positive cells and myeloperoxidase gene expression decreased in older patients with NE-NP, but not in control subjects, CRSsNP, and E-NP. Neutrophil-associated cytokines including IL-17A and IL-23, were negatively correlated with age in NE-NP at the protein and mRNA levels. Additionally, the expression of CXCR2, a receptor for CXCL-1 and CXCL-2, was decreased with age in NE-NP. However, there were no age-related changes in blood neutrophil count, and neutrophil-recruiting chemokines such as CXCL-1, CXCL-2, and CXCL-8. Elderly NE-NP patients showed better endoscopic scores at 12 months after surgery compared with the non-elderly.

**Conclusion:**

Age-related decline in neutrophil inflammation may favorably affect postoperative results in elderly patients with NE-NP.

## Introduction

Recent research using both animal models and human subjects suggests that there are several important changes in the innate and adaptive immune responses with increasing age [1]. Alterations of immune response with aging may affect the pathophysiology of airway inflammation including asthma [2]. We previously reported age-related changes in Western patients with nasal polyps (NP) [3, 4]. In that study, there was a significant age-related decline of eosinophilic inflammation and innate immune barrier function in patients with chronic rhinisinusitis with NP (CRSwNP). Altered barrier function such as decreased S100A8/9 and increased soluble gp130 may be associated with disease extent or asthma comorbidity in eosinophilic NP (E-NP). However, several studies have shown that the inflammatory response in NP removed during surgery is usually eosinophilic in the US and Europe, although the incidence of E-NP is likely overestimated since the population of these study is based on tertiary referral hospital, while NP removed from patients in Asian countries (including China, Korea and Japan) and even from 2^nd^ generation Asians in the US, have inflammation that is much more often non-eosinophilic [5–7]. These two subtypes of NP show different levels of inflammatory cell accumulation and remodeling pattern. Neutrophilic infiltration in non-eosinophilic NP (NE-NP) is often associated with glandular hypertrophy and subsequent fibrosis whereas eosinophils induce edematous changes [8]. NE-NP showed different immunologic characteristics and pathologic mechanism compared with E-NP [9–12] so that age-related immunologic changes would affect disease progression in a different manner. However, up to date, age-related immunologic changes and its clinical implication remained poorly understood in NE-NP. Therefore, the investigation of age-related differences in NE-NP may provide novel clinical implications to clinicians who are treating NP.

## Materials and Methods

### Subjects

One hundred fifty-three study subjects were studied, including 20 controls who underwent sinonasal surgery for unrelated reasons (e.g., endoscopic skull base surgery) without a history of nasal diseases and 133 chronic rhinosinusitis (CRS) patients. Patients were enrolled based on medical chart review (Table 1). CRS diagnoses were based on personal medical history, physical examination, nasal endoscopy, and CT findings of the sinuses according to the European position paper on rhinosinusitis and nasal polyps (EPOS) 2012 guidelines [13]. The presence of NP was confirmed by endoscopic examination. The Lund-MacKay sinus CT scoring system was used as an objective measure of the severity of the disease [14]. The diagnosis of asthma and aspirin sensitivity was performed by an allergist based on history taking, lung function and challenge tests. Aspirin sensitivity was excluded from this study. We evaluated nasal tissue samples such as uncinate process (UP) tissues or NP tissues from patients with CRS without NP (CRSsNP) or CRSwNP, or control subjects. Patients who had taken oral or topical steroids and oral antibiotics within 4 weeks prior to sample collection were excluded from this study. NP were divided into E-NP and NE-NP depending on whether the tissue eosinophils exceeded 10% of the total inflammatory cells [7, 15]. Twenty-eight subjects with E-NP and 20 patients with NE-NP were followed up to out-patient clinic at 12 months after surgery. They underwent endoscopic examination for evaluating Lund-Kennedy endoscopic scores [16]. All patients provided written informed consent, and this study was approved by the Institutional Review Board of Boramae Medical Center, Seoul, Korea.

**Table 1:** Demographic and Clinical characteristics of patients with nasal polyps.

	Control-UP (N = 20)	CRSsNP-UP (N = 63)	CRSwNP-UP (N = 70)	NE-NP (N = 37)	E-NP (N = 43)
**Demographic data**					
Male/Female	14/6	39/24	54/16	30 /7	34 /9
Age (y), mean (SD)	44.3 (19.8)	47.7 (14.0)	50.0 (14.3)	48.7 (15.6)	51.1 (11.9)
Atopy (%)	0 (0)	12 (19)	28 (40)	8 (22)	20 (47)
Asthma (%)	0 (0)	0 (0.0)	4 (5.7)	0 (0.0)	4 (9.3)
Lund-Mackay CT scores	0 (0)	8.6 (5.0)	15.3 (5.1)	15.6 (4.8)	15.1 (5.4)
**Tissue used Methods used**	UP	UP	UP	NP	NP
Tissue homogenate	8	8	26	17	19
Tissue mRNA	20	60	65	34	37
IHC	18	27	65	27	34

UP, uncinated process tissue; NP, nasal polyps; CRSsNP, chronic rhinosinusitis without nasal polyps; CRSwNP, chronic rhinosinusitis with nasal polyps; NE-NP, non-eosinophilic nasal polyps; E-NP, eosinophilic nasal polyps

### Immunohistochemical staining (IHC)

Initially, the authors investigated the influence of age on inflammatory cell distribution in CRS subjects, using IHC. IHC staining was performed by using the polink-2 plus polymerized horseradish peroxidase (HRP) broad DAB Detection System (Golden Bridge International Labs, WA). Briefly, after deparaffinization, the sections were incubated in 3% hydrogen peroxide for endogenous peroxidase inhibition and microwave-treated in 10 mmol/L citrate buffer (pH 6.0) for heat-induced epitope retrieval. The sections were incubated for 60 min at room temperature with each primary antibody, which included mouse anti-human eosinophil major basic protein (EMBP; 1:100; Merck Millipore, Darmstadt, Germany), mouse anti-human mast-cell tryptase (1:500; Abcam, Cambridge, UK), and anti-human neutrophil elastase (HNE) (1:100; Abcam, Cambridge, UK). A proteinase treatment with 0.1% trypsin for 15 minutes in water was performed prior to blocking and antibody staining, particularly during EMBP staining. The sections were incubated in broad antibody enhancer and polymer-HRP for rabbit and mouse antibodies and then stained with the DAB Detection System. Finally, slides were counterstained with hematoxylin. The positive cells in epithelia, glands, and submucosa were counted in the densest five visual fields (400×) by two independent observers, and the average of the resulting scores was used.

### Quantitative Real-time RT-PCR

To investigate changes in the cytokine milieu corresponding to altered immune cell distribution, we used real-time reverse transcription-PCR (qRT-PCR) analysis to measure the mRNA levels of major cytokines and mediators in nasal tissues. The mRNA levels of IL-5, interferon (IFN)-γ, IL-17A, IL-23p19, CXCL-1, CXCL-2, CXCL-8, CCL-11, eosinophil cationic protein (ECP), and myeloperoxidase (MPO) in nasal tissues were evaluated using qRT-PCR analysis as previously described [14]. Briefly, total RNA was extracted from tissue samples with TRIzol reagent (Invitrogen, Carlsbad, CA, USA). One microgram of total RNA was then reverse-transcribed into cDNA using a cDNA Synthesis Kit (amfiRivert Platinum cDNA Synthesis Master Mix, GenDEPOT, Barker, TX, USA). Quantitative RT-PCR was performed with LightCycler 480 SYBR Green I Master mix (Roche, Mannheim, Germany) using specific primers. The primer sequences were as follows: GAPDH, 5´-CATGGGTGTGAACCATGAGAA-3´ for the forward primer and 5´-GGTCATGAGTCCTTCCACGAT-3´ for the reverse primer; eosinophil cationic protein 5´-TCGGAGTAGATTCCGGGTG-3´ for forward primer and 5´-GAACCACAGGATACCGTGGAG-3´ for the reverse primer. In addition, for analysis of IL-5 (Hs01548712_g1), IFN-γ (Hs00989291_m1), IL-17A (Hs00174383_m1), IL-23p19 (Hs00900828_g1), CXCL-1 (Hs00236937_m1), CXCL-2 (Hs00601975_m1), CXCL-8 (Hs00174103_m1), CCL-11(Hs00237013_m1), CCL-24 (Hs00171082_m1), and myeloperoxidase (MPO, Hs00924296_m1), pre-developed assay reagent kits of primers and probes were purchased for TaqMan assays (Life Technologies Korea, Seoul, Korea). Cycling conditions were 95°C for 5 min, followed by 60 cycles at 95°C for 15 sec, 55°C for 20 sec, and 72°C for 20 sec. For normalization, we measured the expression of the GAPDH housekeeping gene. Data were analyzed with Sequence Detection Software version 1.9.1 (Applied Biosystems, Foster City, CA, USA). Relative gene expression was calculated relative to control tissue, using the comparative 2^-ΔΔCT^ method.

### Measurement of cytokine protein levels in tissue homogenates

The protein concentrations for tissue extracts were determined using the Pierce 660nm Protein Assay Kit (Thermo Scientific Inc., NY, USA). Samples were thawed at room temperature and vortexed to ensure well-mixed sample. Multiple cytokine analysis kits (IL-5, IL-17A, IL-23, IFN-γ, CXCL-8, and CCL-11) were obtained from R&D systems (Cat. No. LMSAHM) and data were collected using Luminex 100 (Luminex, Austin, TX, USA). Data analysis was performed using the MasterPlex QT version 2.0 (MiraiBio, Alameda, CA). All assays were run in duplicate according to the manufacturers’ protocol. Sensitivity of each cytokine is as follows: IL-5 (0.5 pg/mL), IL-17A (1.8 pg/mL), IL-23 (11.4 pg/mL), IFN-γ (0.4 pg/mL), CXCL-8 (1.8 pg/mL), and CCL-11 (14.6 pg/mL), All the protein levels in tissue homogenate were normalized to the concentration of total protein (mg/mL) [17].

### Statistical analysis

Statistical analyses were performed using GraphPad Prism 6.0 (GraphPad Software Inc., La Jolla, CA, USA). In this study, the post hoc two-tailed Mann-Whitney *U*-test for unpaired comparisons was used. Test for normality was confirmed by Kolmogorov-Smirnov test. The Spearman correlation coefficient was utilized to determine variable relationship because the data were not normally distributed. Partial correlation analysis was also performed to measure the degree of association between postoperative endoscopic score and age when the effect of infiltrated neutrophils was eliminated. A *P* value of less than 0.05 was considered statistically significant.

## Results

### Age-related decline of neutrophilic infiltration in non-eosinophilic nasal polyps

**Fig 1:**
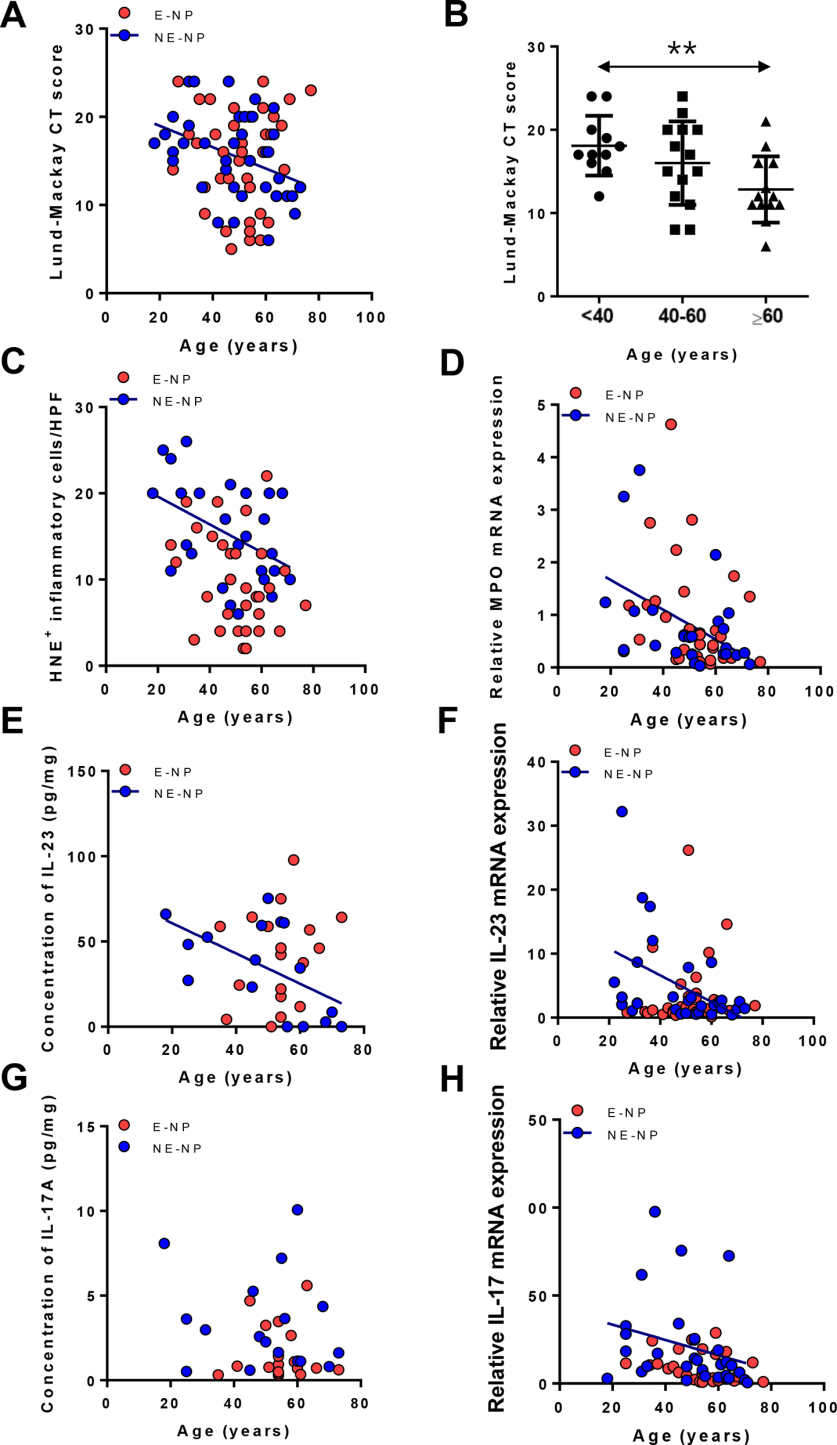
Age-related changes of CT score, neutrophils and neutrophil-associated cytokine in the subtype of nasal polyps. (A) CT score (n = 80). (B) Comparison of CT score among age groups in NE-NP (n = 37, **P<0.01). (C) Human Neutrophil Elastase (HNE)-positive cells (n = 57). (D) Expression of Myeloperoxidase (MPO) mRNA (n = 61). (E) IL-23 protein levels in homogenate (n = 36). (F) Expression of IL-23p19 mRNA (n = 70). (G) IL-17A protein levels in homogenate (n = 36). (H) Expression of IL-17A mRNA (n = 60). E-NP: Eosinophilic nasal polyps; NE-NP: Non-eosinophilic nasal polyps.

In Korean population, age-related decline of CT scores was observed (Fig 1A: R = -0.3921, *P*<0.05).When each group was divided into young-aged (18–39), middle-aged (40–59) and elderly (60–81) groups, the elderly group of NE-NP had significantly lower CT scores than non-elderly one (Fig 1B: *P*<0.01). To investigate age-related changes in immune cell distribution, the correlation analysis between the number of infiltrating inflammatory cells and age was performed. The decreased number of HNE-positive neutrophils was observed in older patients with NE-NP (Fig 1C, R = -0.3881, *P*<0.05), whereas there was no correlation between number of neutrophils and aging in E-NP. Additionally, EMBP-positive and tryptase-positive cells and eosinophil-associated cytokines showed no age-related difference in all nasal tissues (S1 Fig). To confirm this finding, expression of MPO mRNA was investigated and found to have significant negative correlation with age in NE-NP (Fig 1D, R = -0.5200, *P*<0.01). HNE-positive cells and MPO expression were statistically significantly decreased in elderly patients with non-eosinophilic NPs (HNE-positive cells per high power field: 16.5±5.8 vs. 12.5±5.0 vs. 11.2±4.5; MPO mRNA expression: 2.2 folds vs. 1.4 folds vs. 1 fold).

### Age-related decline of neutrophil-associated cytokines in non-eosinophilic nasal polyps

**Fig 2:**
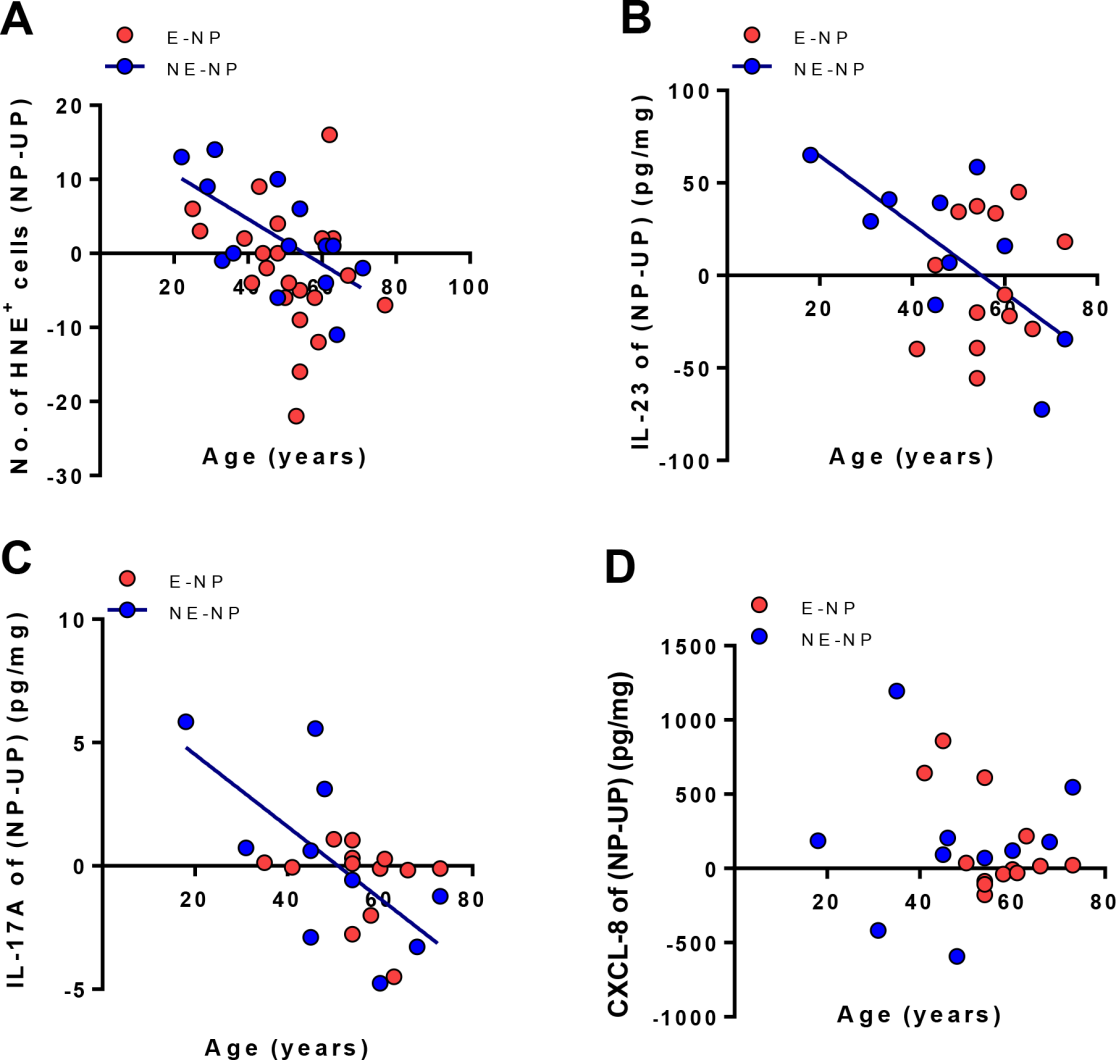
Age-related changes of neutrophils and neutrophil-associated cytokines between nasal polyps and uncinated process tissues. (A) Human Neutrophil Elastase (HNE)-positive cells (n = 38). (B) IL-23 protein levels in homogenate (n = 23). (C) IL-17A protein levels in homogenate (n = 23), (D) CXCL-8 protein levels in homogenate (n = 23). Y-axis means the values subtracting levels of uncinate process tissue from those of nasal polyps from the same patient (NP-UP). E-NP: Eosinophilic nasal polyps; NE-NP: Non-eosinophilic nasal polyps.

Up-regulation of cytokines that neutrophils produce or response to [18], including IL-17A and IL-23, was negatively correlated with age in NE-NP at the protein or mRNA levels (Fig 1E, IL-23 protein, R = -0.5756, *P*<0.05; Fig 1F, IL-23p19 transcript, R = -0.3946, *P*<0.05; Fig 1H, IL-17A transcript, R = -0.4165, *P*<0.05), whereas there was no correlation in E-NP and UP from controls (data not shown) and CRS subjects (Fig 1 and S2 Fig). However, protein levels of IL-17A did not show age-related decline (Fig 1G), which might be affected by small number of study population. Therefore, authors took UP tissue, which is the ethmoidal mucosa near the site at which polyps form, and NP tissue from the same patient, then, subtracted cytokine values of UP from those of NP (NP-UP) to examine the decline of IL-17A and IL-23 with age. Interestingly, these subtracted values were decreased to minus with aging in neutrophils and its associated cytokines (Fig 2A, HNE-positive cells, R = -0.5973, *P*<0.05; Fig 2B, IL-23 protein, R = -0.6485, *P*<0.05; Fig 2C, IL-17A protein, R = -0.6444, *P*<0.05). These findings imply that neutrophilic decline with aging was prominent especially in NE-NP tissues compared to other nasal tissues.

### No age-related changes in blood neutrophil and neutrophil-recruiting chemokines

**Fig 3:**
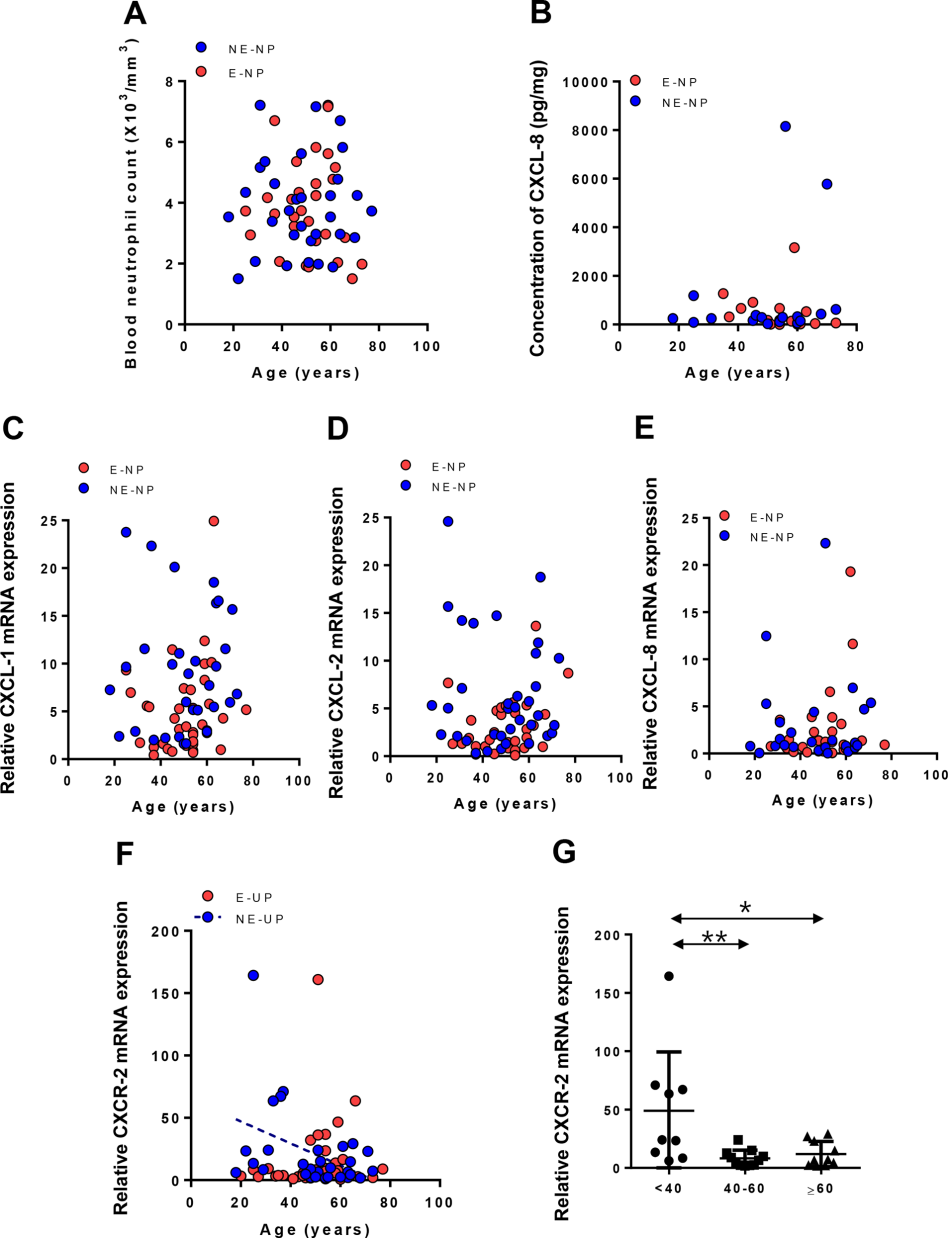
Age-related changes of blood neutrophils, neutrophil-recruiting chemokines and their receptor in the subtype of nasal polyps. (A) Blood neutrophil count (n = 61). (B) CXCL-8 protein levels in homogenate (n = 36). (C) Expression of CXCL-8 mRNA (n = 54). (D) Expression of CXCL-1 mRNA (n = 70). (E) Expression of CXCL-2 mRNA (n = 70). (F) Decline of expression of CXCR-2 mRNA in NE-NP (n = 70; R = -0.323, *P* = 0.08). (G) Comparison of expression of CXCR-2 mRNA among age groups in NE-NP (n = 70, *P<0.05; **P<0.01), E-NP: Eosinophilic nasal polyps; NE-NP: Non-eosinophilic nasal polyps.

To elucidate the mechanism of the age-related decline of neutrophils in NE-NP, the number of neutrophils in circulating blood, neutrophil-attracting chemokines such as CXCL-1, CXCL-2 and CXCL-8, and CXCR-2, a receptor for CXCL-1 and CXCL-2 in NP tissue were analyzed. Interestingly, there were no age-related differences in peripheral blood neutrophil counts and chemokine levels in subjects with CRS with NP (Figs 2D and 3A–3E), but age-related difference exists in the expression of CXCR2 in NE-NP, but not in E-NP (Fig 3F and 3G).

### Postoperative endoscopic score has negative correlations with age in NE-NP

**Fig 4:**
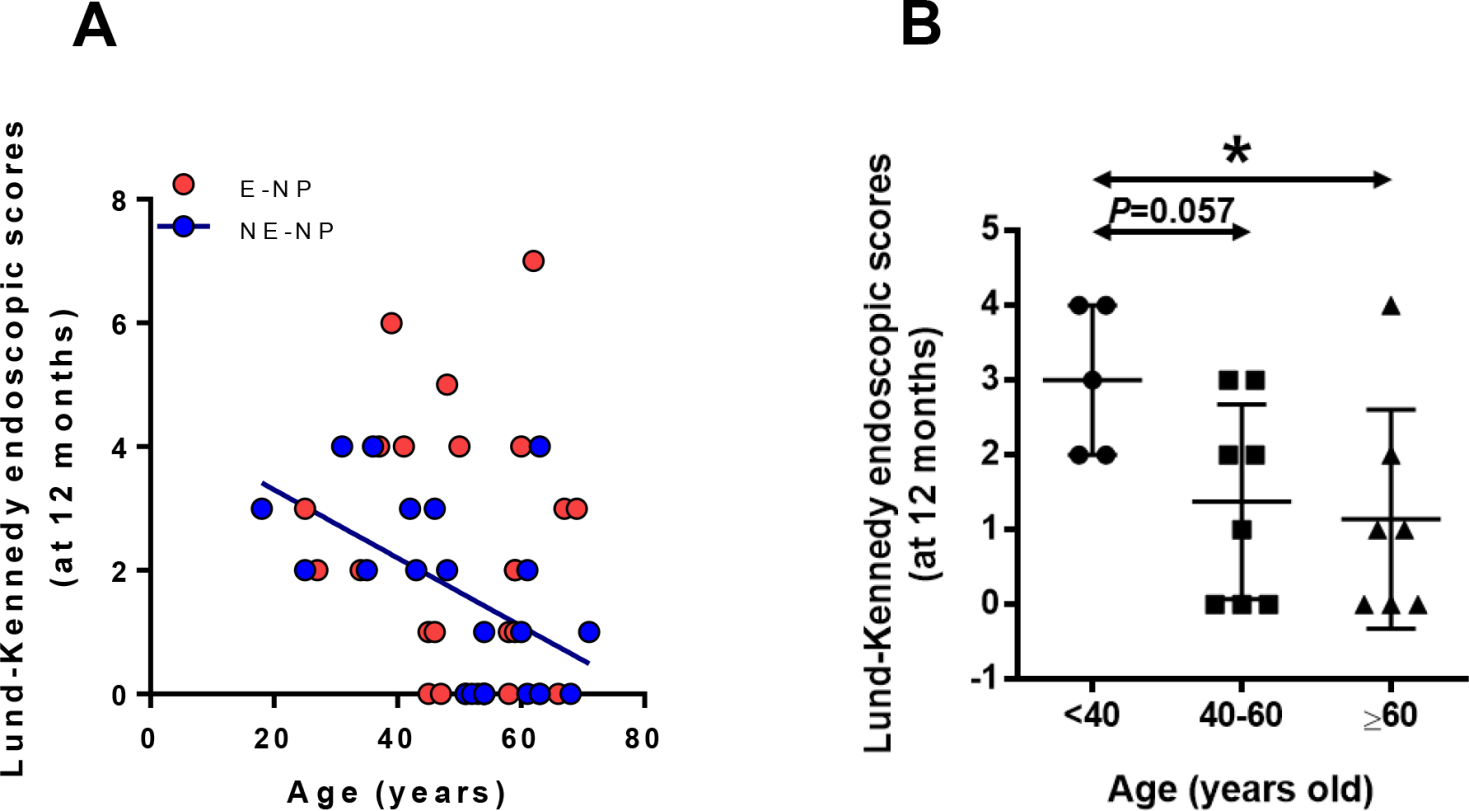
Age-related changes of postoperative endoscopic scores. (A) Lund-Kennedy endoscopic scores (n = 48). (B) Comparison of endoscopic scores among age group in NE-NP (n = 20, *P<0.05).

To investigate the clinical impact of the age-associated decline of neutrophils in NE-NP, the association of postoperative prognosis with age, using Lund-Kennedy endoscopic score, was assessed. Postoperative endoscopic score showed significant negative correlations with age (Fig 4A, R = -0.5550, *P*<0.05). When each group was divided into young-aged (18–39), middle-aged (40–59) and elderly (60–81) groups, postoperative endoscopic score was statistically significantly decreased in elderly patients with non-eosinophilic NPs (Fig 4B, *P*<0.01). Partial correlation analyses were performed with statistical adjustment of the number of HNE positive cells to investigate their correlation when the effect of neutrophils was eliminated. Postoperative endoscopic scores were no longer significantly related to age (data not shown). These findings suggest that the decline of neutrophils with age may influence on endoscopic scores related to postoperative prognosis.

## Discussion

We recently reported that elderly CRS patients have higher CT scores and show higher prevalence of asthma in a group of patients in Chicago, which would be mainly eosinophilic NPs [3, 4]. In contrast to NPs of Western countries, earlier studies reported that elderly NPs showed better objective surgical outcomes and lower epithelial proliferative ability, compared with younger age groups in Korean population [19, 20]. However, previous study design had a limitation that the subjects were not divided into each subtype of CRS and they did not investigate age-associated immune changes. For the first time, this study showed that neutrophilic infiltration was decreased with aging in NE-NP. Considering that neutrophil numbers in the peripheral blood and neutrophil-recruiting chemokines in the polyp tissues did not vary with age, aging may affect neutrophil survival or responsiveness rather than recruitment. CXCL-8-induced activation of neutrophils causes delayed apoptosis and enhanced production of cytokines and reactive oxygen species [21]. However, CXCL-8-stimulated neutrophils from the elderly did not show enhanced MPO and elastase activity, meaning poor responsiveness to CXCL-8 in elderly neutrophils [22]. Additionally, neutrophils from the elderly subjects were less protected from apoptosis when stimulated with LPS, IL-2 and GM-CSF, than neutrophils from younger ones [23]. In line with previous reports, neutrophil-producing enzymes such as MPO and neutrophil-associated cytokines such as IL-17A and IL-23 were significantly decreased with aging, although there was no difference in the expression of CXCL-8 in this study. Functional defect of neutrophils in the elderly NE-NP may be associated with inflammatory involution, taking it into account that neutrophils are one of main inflammatory cells in NE-NP.

Recent study using a large all-payer database showed that patients >65 were three times more likely to have a major complication of endoscopic sinus surgery such as skull base, orbital and hemorrhagic injuries, compared to younger adults [24]. Additionally, elderly patients usually have more complicated underlying diseases and need more time to restore to normal life after surgery. The optimal surgical management of nasal polyps has not yet been established [13]. The efficacy of procedures may well be dependent on a variety of factors including disease extent and immunologic characteristics. Therefore, the specific details of the procedures performed need be considered carefully. Complete "full house" surgery can be recommended for nasal polyposis if the elderly patients have no comorbidities in general. However, from our data showing spontaneous involution pattern of disease extent and neutrophilic infiltration in the elderly patients with NE-NP, strategy for maximal medical treatment may be an option in these elderly NE-NP patients who are at high risk for surgical complication. Moreover, based on our data demonstrating a better postoperative course in elderly patients with NE-NP compared to the non-elderly, strategy for “minimally invasive surgery such as simple polypectomy” might be suggested in elderly NE-NP patients who have high risk for long-time surgery due to medical conditions such as heart diseases.

Lastly, this study failed to demonstrate age-related changes in E-NP in Korean population, while previous studies showed that elderly patients with Western NP, majority of which is E-NP, have higher prevalence of asthma and CT scores regardless of decreased ECP levels [3, 4]. Possible explanation is that E-NP in Asian population showed somewhat different characteristics compared to Western E-NP. Previous studies demonstrated asthma prevalence of 4 to 26.9% and atopy of 21 to 44.3% in E-NP of East Asia, compared with over 50% asthma and atopy rates in the U.S. [7, 8, 25, 26]. Majority of Asian NP is non-eosinophilic. Furthermore, E-NP in Asians seems less eosinophilic and less prevalent comorbidities of allergic diseases than those in Westerns, implying a different or less severe inflammatory mechanism compared with Western NP. Our study population showing asthma rate of 9.3% and atopy of 47% is consistent with previous Asian reports. Therefore, further investigation would be valuable regarding the endotype of E-NP according to geographic distribution.

In summary, this study demonstrates that elderly patients with NE-NP have lower number of neutrophilic infiltration and lower expression neutrophil-associated cytokines compared with the non-elderly. These findings correlated with better postoperative course.

## Supporting Information

S1 Fig Age-related changes of eosinophils and mast cells in the subtype of nasal polyps. (A) Eosinophilic major basic protein (EMBP)-positive cells (n = 57). (B) IL-5 protein levels in homogenate (n = 36). (C) CCL-11 protein levels in homogenate (n = 36). (D) Blood eosinophil count (n = 61). (E) Tryptase-positive cells (n = 52). E-NP: Eosinophilic nasal polyps; NE-NP: Non-eosinophilic nasal polyps
(TIF)

S2 Fig Age-related changes of blood neutrophils and neutrophil-recruiting chemokines in uncinate process tissues from patients with CRSsNP and CRSwNP. (A) Human Neutrophil Elastase (HNE)-positive cells (n = 68). (B) expression of Myeloperoxidase (MPO) mRNA (n = 74). (C) IL-17A protein levels in homogenate (n = 34). (D) Expression of IL-17A mRNA (n = 77). (E) IL-23 protein levels in homogenate (n = 34). (F) Expression of IL-23p19 mRNA (n = 84).
(TIF)
