# Supplementary figures and images for: Age-Related Decline of Neutrophilic Inflammation Is Associated with Better Postoperative Prognosis in Non-eosinophilic Nasal Polyps
Source: PLoS One. 2016 Feb 5;11(2):e0148442. doi: 10.1371/journal.pone.0148442 (PMC4743847; doi:10.1371/journal.pone.0148442)

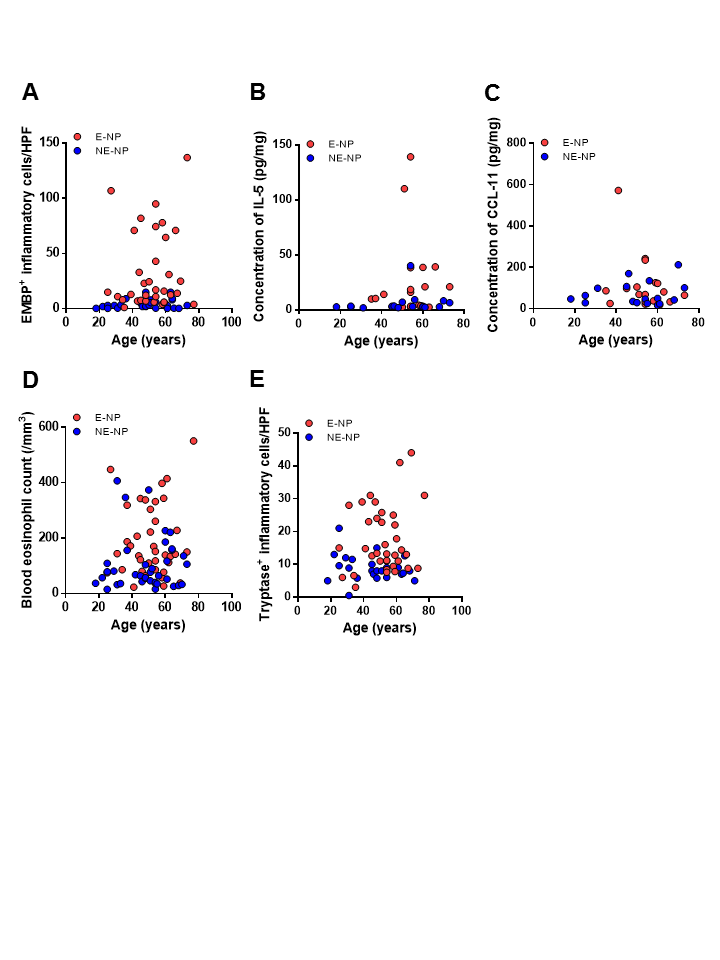

Supplement: S1 Fig — (A) Eosinophilic major basic protein (EMBP)-positive cells (n = 57). (B) IL-5 protein levels in homogenate (n = 36). (C) CCL-11 protein levels in homogenate (n = 36). (D) Blood eosinophil count (n = 61). (E) Tryptase-positive cells (n = 52). E-NP: Eosinophilic nasal polyps; NE-NP: Non-eosinophilic nasal polyps (TIF) [file pone.0148442.s001.tif]

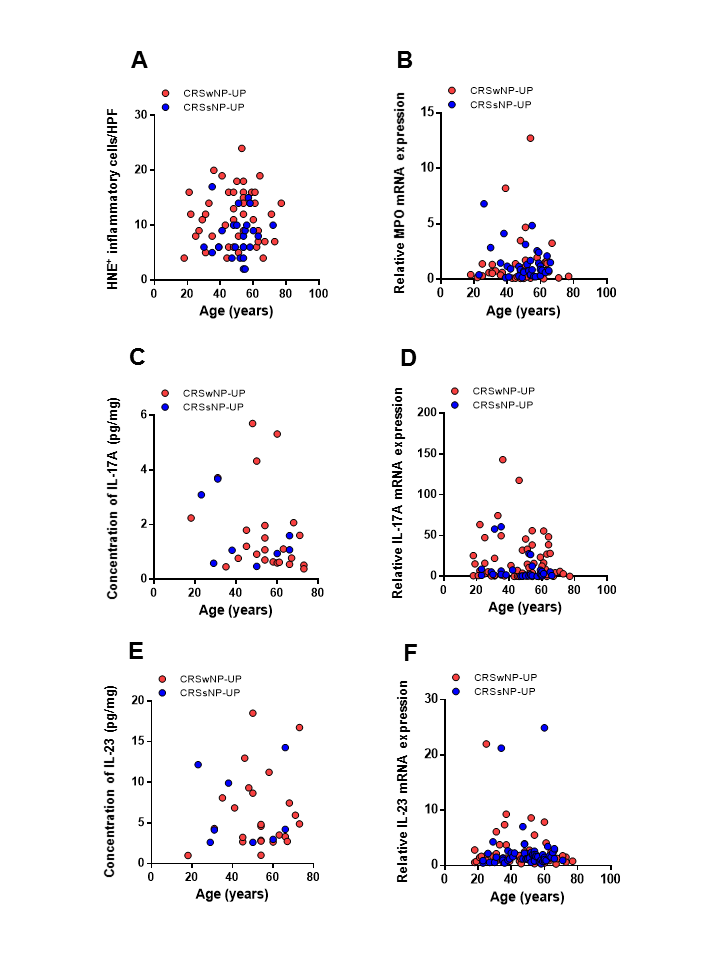

Supplement: S2 Fig — (A) Human Neutrophil Elastase (HNE)-positive cells (n = 68). (B) expression of Myeloperoxidase (MPO) mRNA (n = 74). (C) IL-17A protein levels in homogenate (n = 34). (D) Expression of IL-17A mRNA (n = 77). (E) IL-23 protein levels in homogenate (n = 34). (F) Expression of IL-23p19 mRNA (n = 84). (TIF) [file pone.0148442.s002.tif]
